# Sex differences in skeletal muscle revealed through fiber type, capillarity, and transcriptomics profiling in mice

**DOI:** 10.14814/phy2.15031

**Published:** 2021-09-21

**Authors:** Juliana O’Reilly, Kikumi D. Ono‐Moore, Sree V. Chintapalli, Jennifer M. Rutkowsky, Todd Tolentino, K. C. Kent Lloyd, I. Mark Olfert, Sean H. Adams

**Affiliations:** ^1^ Division of Exercise Physiology West Virginia University School of Medicine Morgantown West Virginia USA; ^2^ Arkansas Children’s Nutrition Center Little Rock Arkansas USA; ^3^ Department of Pediatrics University of Arkansas for Medical Sciences Little Rock Arkansas USA; ^4^ Department of Molecular Biosciences, University of California Davis School of Veterinary Medicine Davis California USA; ^5^ Mouse Metabolic Phenotyping Center University of California Davis California USA; ^6^ Mouse Biology Program University of California Davis California USA; ^7^ Department of Surgery University of California Davis School of Medicine Sacramento California USA; ^8^ Center for Alimentary and Metabolic Science University of California Davis School of Medicine Sacramento California USA

**Keywords:** muscle performance, myocyte, neovascularization, sexual dimorphism

## Abstract

Skeletal muscle anatomy and physiology are sexually dimorphic but molecular underpinnings and muscle‐specificity are not well‐established. Variances in metabolic health, fitness level, sedentary behavior, genetics, and age make it difficult to discern inherent sex effects in humans. Therefore, mice under well‐controlled conditions were used to determine female and male (*n* = 19/sex) skeletal muscle fiber type/size and capillarity in superficial and deep gastrocnemius (GA‐s, GA‐d), soleus (SOL), extensor digitorum longus (EDL), and plantaris (PLT), and transcriptome patterns were also determined (GA, SOL). Summed muscle weight strongly correlated with lean body mass (*r* ^2^ = 0.67, *p* < 0.0001, both sexes). Other phenotypes were muscle‐specific: e.g., capillarity (higher density, male GA‐s), myofiber size (higher, male EDL), and fiber type (higher, lower type I and type II prevalences, respectively, in female SOL). There were broad differences in transcriptomics, with >6000 (GA) and >4000 (SOL) mRNAs differentially‐expressed by sex; only a minority of these were shared across GA and SOL. Pathway analyses revealed differences in ribosome biology, transcription, and RNA processing. Curation of sexually dimorphic muscle transcripts shared in GA and SOL, and literature datasets from mice and humans, identified 11 genes that we propose are canonical to innate sex differences in muscle: *Xist*, *Kdm6a*, *Grb10*, *Oas2*, *Rps4x* (higher, females) and *Ddx3y*, *Kdm5d*, *Irx3*, *Wwp1*, *Aldh1a1*, *Cd24a* (higher, males). These genes and those with the highest “sex‐biased” expression in our study do not contain estrogen‐response elements (exception, *Greb1*), but a subset are proposed to be regulated through androgen response elements. We hypothesize that innate muscle sexual dimorphism in mice and humans is triggered and then maintained by classic X inactivation (*Xist*, females) and Y activation (*Ddx3y*, males), with coincident engagement of X encoded (*Kdm6a*) and Y encoded (*Kdm5d*) demethylase epigenetic regulators that are complemented by modulation at some regions of the genome that respond to androgen.

## Graphical abstract

Anatomical and molecular phenotyping of mouse muscle illustrates that there are innate differences between males and females. Gene expression profiling, in particular, revealed hundreds to thousands of differentially abundant transcripts between the sexes, with muscle group‐specific patterns comparing gastrocnemius ("GASTROC") and soleus.

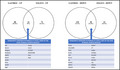

## 1 INTRODUCTION

The molecular underpinnings of sex‐specific functional and metabolic characteristics in skeletal muscle remain to be fully elaborated. Furthermore, identifying innate sexually dimorphic systems—vs. those that are adaptive to changes in fitness, age, environment, or other factors—is of interest. There are many examples of sexually dimorphic differences in human muscle function, including strength and fatigue. For instance, leg and arm muscle strength in middle‐aged to elderly adults were ~37–58% higher in males (Frontera et al., 1985) and qualitatively this was also seen in men in their early 20s (Miller et al., 1993). Similarly, isometric and concentric peak torque (N⋅m) values associated with knee extensions were ~60% higher in men compared to women (Yasuda et al., 1985). Strength gain and myofiber hypertrophy were significantly greater in men compared to women after knee extensor resistance training for 26 week (Bamman et al., 2003). During short‐term “burst” type exercise, men had significantly faster starting speed rates in a 5 or 20 s maximum cycling sprint test paradigm (Billaut and Bishop, 2012; Wiecek et al., 2016), displayed greater mean power (by ~26–48%) or peak power (by ~31–44%) in a Wingate 30 s cycle sprint test (Esbjornsson et al., 1993; Sbriccoli et al., 2007), and had a quicker time to peak power and ~55% higher power measures in a different cycling sprint test (Wiecek et al., 2016). Fatigue outcomes generally support the idea that females have a higher capacity to maintain performance and with a faster recovery following challenges that elicit significant muscle work. For example, in a study of strength‐matched men and women there was a smaller decline in force‐generating capacity over time of the adductor pollicis muscle (intermittent static contractions) in women, and time to exhaustion was ~15 min in women when compared to ~8 min in men (Fulco et al., 1999). In a cycling sprint test to determine maximum and sustained power followed by power decline (W/kg⋅s), the latter was lower by ~20% in young women compared to men, indicative of greater anaerobic endurance in women (Wiecek et al., 2016). With repetitive leg extensor loading (squat exercises), maximal isometric force dropped less in females (by 29%) compared to males (by 47%), and maximal force recovery with rest was relatively higher in women (Hakkinen, 1994). Billaut and Bishop (2012) also reported a lower relative mechanical work decrement over time in female athletes compared to male athletes, when using a test of 20 replicates of 5 s cycle sprints. Interestingly, however, relative decrements across all men and women closely correlated with initial single‐sprint mechanical work achieved (*r* = 0.89), and a post hoc analysis comparing a subset of initial work‐matched men and women erased sex differences in work decrement (Billaut and Bishop, 2012). Taken together, results on muscle function and fatigue indicate that certain phenotypes have inherent sex differences whereas others are modifiable and responsive to fitness or training irrespective of sex.

Sexual dimorphism in performance and fatigue may be driven by differences in myofiber characteristics, muscle size or other factors such as blood supply (capillarity). Strength indices such as single “rep” maximum force (1RM, in Newtons) for leg or arm resistance exercise are tightly correlated to the whole muscle cross‐sectional area (CSA), typically higher in males (Miller et al., 1993). In adults aged 61 to 77 years, myofiber CSAs of all types (type I, IIa, IIx) were on average 39–42% higher in males (Bamman et al., 2003). Similar findings are consistently reported in young adults, with the caveat that myofiber CSAs are highly variable across individuals and fiber types (Hoeg et al., 1985; Miller et al., 1993; Porter et al., 1985; Simoneau and Bouchard, 1989; Staron et al., 2000; Yasuda et al., 1985). With respect to percentages of myofiber sub‐types, data are mixed and may be muscle group‐specific. In one study, women had a higher prevalence of "oxidative” type I fibers (~44% vs. ~36% in males) and lower prevalence of “glycolytic” type II fibers in vastus lateralis (e.g., ~34% vs. ~41% in males) (Staron et al., 2000). This finding in vastus lateralis was also observed by Miller et al. (1993) but no difference was seen in biceps brachii. Sex differences in percentage fiber type are not always present, however, and as with myofiber CSA there is tremendous person‐to‐person variability (e.g., Porter et al., 1985; Simoneau and Bouchard, 1989; Yasuda et al., 1985). In addition to sexual dimorphism in whole‐muscle and myofiber cross‐sectional areas, and possible differences in fiber type prevalence, other factors that could modify performance and fatigue include muscle bed O_2_ supply (e.g., capillarity). At least two studies have demonstrated no significant differences in muscle capillary density comparing men and women (Porter et al., 1985; Sjogaard, 1982), in contrast to other groups that reported a ~25–30% higher vastus lateralis capillary density in women (Hoeg et al., 1985; Roepstorff et al., 2006). Thus, whether or not there is sexual dimorphism in skeletal muscle capillary density remains an open question.

Gaining a fuller understanding of sex differences in muscle function and metabolism is important to optimize human performance and health, and to consider science‐based strategies for healthy aging and sarcopenia prevention. There is consensus that muscle size and performance differ between the sexes in humans, vis‐à‐vis muscle cross‐sectional area, strength, force generation, burst speed, and fatigue. Nevertheless, for myofiber type prevalence, myofiber size, and muscle capillarity, substantial population variability is evident which makes it challenging to uncover sex‐specific impacts on these parameters. Considering results from human twin studies and rodent genetics, Simoneau and Bouchard (1989) postulated that fiber type distribution is primarily regulated by non‐genetic factors. At a minimum, fitness level must be accounted for when considering muscle performance and metabolism (Beever et al., 1985; Billaut and Bishop, 2012), but other factors could play a role (e.g., diet, genetic admixture, environment) and can be challenging in human studies. Animal models have the advantage of precise control over diet, environment, age, and genetics, which enables a comprehensive assessment of innate sex differences in muscle fiber type, capillarity, and molecular phenotypes, including the transcriptome. As for the latter, there have been surprisingly few published reports in this regard, i.e., transcript profiling of murine mixed muscle by Yang et al. (2006) and Yoshioka et al. (2007), respectively, with the latter group only comparing one pooled sample per sex. To our knowledge, there have been no published studies focusing on sexual dimorphism across disparate muscle types. Previous studies also did not have the advantage of applying RNASeq technology and the full murine genomic sequence to enable the broadest possible transcriptomics coverage and gene annotation. This prompted the current set of experiments to determine and compare myofiber, capillarity, and transcriptomics variables across mixed muscle (gastrocnemius) and myoglobin‐rich soleus muscle in male and female mice.

## 2 MATERIALS AND METHODS

### 2.1 Animals

All in vivo studies were completed by the University of California (UC) Davis Mouse Biology Program and the Energy Balance, Exercise and Behavior Core of the NIH/NIDDK‐funded UC Davis Mouse Metabolic Phenotyping Center. Experiments were approved by the UC Davis Institutional Animal Care and Use Committee (Protocol #20396). Adult ~19 week old female (*n* = 19) and male (*n* = 19) C57BL/6Ncrl mice wildtype littermates from heterozygous breeding of C57BL/6NCrl‐*Mb^em3Shad^*/Mbp and C57BL/6NCrl‐Mb^em2Shad^/Mbp mice were used to study skeletal muscle phenotypes. We have previously reported on metabolic phenotypes in response to high‐fat feeding and cold challenges in a subset of these animals (9 wildtype [WT] females, 10 WT males; C57BL/6NCrl) that were compared to myoglobin knockout littermates (C57BL/6NCrl‐*Mb^em3Shad^*) (Ono‐Moore et al., 2021). To evaluate sexually dimorphic muscle phenotypes using a more robust sample size, herein we leveraged the muscle samples from the prior experiment plus additional WT mice under identical experimental conditions (including the high‐fat diet paradigm). Briefly, mice were individually housed in positively‐ventilated duplex cages (allowing visual and scent cues to the neighboring mouse). Four independent cohorts totaling 19 males and 19 females weaned at 3 week of age were tested. Other than periods in which indirect calorimetry was performed, mice were housed in a vivarium maintained at 20–24°C and 30–40% relative humidity with a 12 h light/12 h dark cycle (lights on, 06:00), under specific pathogen‐free housing conditions with water and food provided ad libitum. At ~4 week of age, mice were acclimated in the facility for 1 week before undergoing baseline CLAMS (Comprehensive Lab Animal Monitoring System, Columbus Instruments, Columbus, OH) indirect calorimetry measurements starting at 5 week of age while being fed a standard chow diet (Teklad 2918, Envigo). After returning to their home cages from the CLAMS, mice were switched to a high‐fat diet (HFD; Teklad 08511, Envigo) providing 45% of energy from fat. Diets were fed for ~13 week, during which animals underwent two additional CLAMS measurements, glucose tolerance and insulin tolerance tests, and cold challenges (Ono‐Moore et al., 2021).

### 2.2 Tissue collection, muscle fiber‐typing, and vascularity

The final CLAMS experiment included an assessment of body composition and bone by dual energy x‐ray absorptiometry (DEXA) under isoflurane anesthesia, using a Lunar PIXImus II Densitometer and the manufacturer’s standard protocol that excludes the head region (GE Medical Systems). The mice were then returned to their home cages, and tissue collections were done ~1 week later. Starting at ~05:00 mice were briefly fasted (~4–6 h, depending on a given animal’s place in the queue) and then euthanized by transthoracic cardiac puncture while anesthetized under isoflurane/O_2_, coincident with cardiac blood collection. White adipose tissues (subcutaneous [between the abdominal wall and epidermal layer], inguinal, retroperitoneal [separated from the perirenal depot], perigonadal) and interscapular brown adipose tissue (BAT) depots were weighed and samples snap frozen in liquid nitrogen and stored at –70°C potential future use. The triceps surae [gastrocnemius (GA), soleus (SOL), and plantaris (PLT)] muscles, and extensor digitorum longus (EDL) muscle were excised from both legs. Heart and left‐side skeletal muscles were weighed and snap frozen in liquid nitrogen. Right‐side skeletal muscles and top half of the heart were embedded and frozen in OCT (optimal cutting temperature) cryosection media. The order of the mice used for tissue and blood collection was randomized across experimental groups.

#### 2.2.1 Tissue preparation for histology

From the right leg, the mid‐belly of each muscle was quickly and carefully excised and placed on a cork (with OCT) and then flash frozen in liquid N_2_ cooled isopentane. The heart was cut across the middle of the left and right ventricles, placed on a cork (with OCT) and then flash frozen in liquid N_2_ cooled isopentane. All samples were then stored at –80˚C until processed for molecular or morphological analyses.

#### 2.2.2 Muscle fiber‐typing and capillarity

Frozen tissue was cut using a –20˚C cryomicrotome (Jung‐Reichert Cryocut 1800; Cambridge Instruments) to yield 8 μm‐thick transverse sections. The cryosectioned muscles were cut along the transverse plane. All tissues were stained for fiber type using the lead nitrate‐ATPase method described by Rosenblatt et al. (1987) allowing for direct assessment of lightly stained (type II) fibers, and darkly‐stained (type I) fibers. Capillarity assessment was done using FITC‐labeled Griffonia isolectin B4 as previously reported (Gorman et al., 2014; Uchida et al., 2015). Here, muscle sections on the slides were fixed in 4% paraformaldehyde and stained with FITC‐conjugated *Griffonia simplicifolia* I isolectin (1:100; Vector Labs) for 30–60 min. Light or fluorescent microscopy (using 40X objective) was used to digitally acquire images of sectioned muscle regions of interest, from each muscle type. Capillary and myofiber counting were performed by a single individual who was blinded to the identity of each of the samples. For the GA and heart muscles, we obtained images in a checkerboard fashion across the entire muscle (endocardial area for heart). The GA muscle was sub‐divided into superficial (GA‐s) and deep (GA‐d) regions to allow for evaluation and accounting of regional differences within the GA (i.e., GA‐s typically comprises nearly 100% type II myofibers, whereas GA‐d has a more heterogeneous mixture of type I & II myofibers). Designation of GA‐s was defined as outer 50% of the GA, while GA‐d was defined as the inner 50% of the muscle section. For the other muscles, full‐screen non‐overlapping images were obtained and analyzed. Counting was performed by visualization from acquired images using NIH Image J software to visually mark/count the capillaries and fibers on each image. Capillary‐to‐fiber ratio (C:F; number of capillaries/number muscle fibers), capillary density (CD; number of capillaries/muscle fiber area), and fiber cross‐sectional area (FCSA) were measured and calculated.

### 2.3 Muscle transcriptomics

RNA was isolated from a single GA and a single SOL muscle per mouse in order to evaluate transcriptomics patterns in each tissue. As previously described in detail (Ono‐Moore et al., 2021), cryo‐pulverized GA powder (~25 mg) or whole SOL were homogenized in Tri Reagent (Invitrogen, ThermoFisher Scientific) then incubated at room temperature for 5 min before being frozen at –80°C. Once thawed, 1‐bromo‐3‐chloropropane (BCP, Sigma) was added (1:100 BCP:Tri Reagent), samples vortexed and then incubated at room temperature for 15 min, and centrifuged at 20,800 g for 15 min at 4°C. The aqueous phase was mixed with equal parts of 70% ethanol and applied to RNeasy mini spin columns (Qiagen). The spin columns were washed once with half the recommended volume of RW1 buffer followed by a DNase treatment (15 min, Qiagen), then washed with half the recommended volume of RW1 buffer. The remaining steps were done per the manufacturer’s protocol. RNA concentrations and 260/280 ratios were determined via UV spec, and quality was assessed using an Agilent TapeStation system.

For transcriptomics analyses of mixed muscle (GA) and type I‐ and Mb‐rich muscle (SOL), next generation sequencing and RNA‐seq library preparations were performed by the UAMS Genomics Core Facility located in the Winthrop P. Rockefeller Cancer Institute at the University of Arkansas for Medical Sciences. RNA‐seq libraries were prepared using the Illumina TruSeq Stranded total RNA Library Prep Kit with TruSeq Unique Dual Indexed adapters. Libraries were assessed for mass with the Qubit 3.0 fluorometer using the Qubit 1X dsDNA High‐Sensitivity Assay Kit (ThermoFisher Scientific), for functionality with the KAPA Library Quantification Kit (Roche Sequencing and Life Science), and for fragment size with the Agilent Fragment Analyzer using the High‐Sensitivity NGS Gel Kit. Molarities were calculated for libraries, and then diluted and denatured according to Illumina recommendations for clustering on a cBot and paired‐end sequencing on a HiSeq 3000 with a 150‐cycle SBS kit for 2 × 75 reads. Alignment to the mouse (GRCm38) genome was carried out using STAR (Dobin et al., 2013). All the aligned reads were exported in BAM format and subsequent data analysis was performed in the SeqMonk software package (Babraham Bioinformatics, Cambridge, UK). Differentially‐expressed genes comparing female and male mice were analyzed using iDEP version 0.92 (http://bioinformatics.sdstate.edu/idep/) (Ge et al., 2018). We used the “Pathway” feature, GAGE method (for “GO Biological Process,” “GO Molecular Function,” “GO Cellular Component,” and “HMDB” modes), with pathway FDR‐adjusted significance cutoff of 0.2, choosing absolute values of fold changes, a minimum/maximum of 15/2000 for geneset size, and showing up to 30 pathways (if relevant).

### 2.4 Statistical analysis

Unless otherwise noted, Student’s *t*‐test was used to assess differences between males and females for any given factor (GraphPad Prism, version 9.0).

## 3 RESULTS

### 3.1 Animal characteristics

Muscle and tissue weights, body weight, and body composition following the ~13 week high‐fat feeding period are presented in Table 1. As expected, body weight and lean mass were higher in male mice and, with the exception of the brain, tissue weights were heavier in males. Muscle weights were uniformly higher in males compared to females, and the summed muscle weight was significantly higher by 18% in males. There were no statistically significant sex differences in bone mineral density or bone mineral content.

**TABLE 1:** Tissue weights, bone parameters and body composition of female and male mice after ~ 13 of weeks on a high‐fat diet

	Females (*n* = 19)	Males (*n* = 19)	*t*‐test^1^
Body weight (g)	26.3 ± 0.85	33.8 ± 0.72	***
Liver (g)	0.98 ± 0.03	1.23 ± 0.03	***
Brain (mg)	486 ± 6.71	458 ± 6.81	*
Heart (mg)	130 ± 2.13	144 ± 3.00	***
Left gastrocnemius (mg)	109 ± 1.94	128 ± 3.15	***
Left plantaris (mg)	13.4 ± 0.40	18.0 ± 0.31	***
Left soleus (mg)	8.40 ± 0.26	9.55 ± 0.72	0.14
Left extensor digitorum longus (mg)	8.23 ± 0.26	9.00 ± 0.34	0.08
Summed left side muscles (mg)	135.2 ± 2.0	159.8 ± 3.9	***
Perigonadal adipose^2^ (mg)	974 ± 120	1815 ± 104	***
Inguinal adipose^2^ (mg)	257.2 ± 27.2	316.3 ± 27.4	0.13
Retroperitoneal adipose^2^ (mg)	254.2 ± 32.6	548.5 ± 32.0	***
Subcutaneous adipose^2^ (mg)	735.2 ± 83.1	1018.0 ± 62.5	*
Brown adipose tissue^2^ (mg)	144.9 ± 11.4	273.6 ± 15.6	***
Summed white adipose^3^ (g)	2.37 ± 0.26	3.97 ± 0.22	***
DEXA fat mass (g)	6.70 ± 0.64	10.97 ± 0.71	***
DEXA lean mass (g)	16.98 ± 0.25	20.44 ± 0.20	***
DEXA bone mineral density (g/cm^2^)	0.0486 ± 0.0003	0.0478 ± 0.0003	0.10
DEXA bone mineral content (g)	0.4372 ± 0.0077	0.4429 ± 0.0128	0.70

Values are means ± SEM.

DEXA measurements were made ~1 week prior to tissue collection.

^1^ Two‐sided *t*‐test, *p < 0.05; ***p < 0.001.

^2^ Sum of left and right depots are reported; BAT is the interscapular depot.

^3^ Sum of perigonadal, inguinal, retroperitoneal, subcutaneous white adipose depots.

#### 3.1.1 Capillary and skeletal muscle fiber type profiles in female and male mice

We have previously published methods and illustrative examples of capillary and fiber histology images (Ono‐Moore et al., 2021); the same approaches were used herein. Indices of capillarity in heart and multiple muscle groups (GA‐superficial [GA‐s], GA‐deep [GA‐d], SOL, EDL, and PLT) are presented in Table 2. These results demonstrate that capillary density (# of capillaries/mm^2^) and capillary:fiber ratio (number of capillaries:number of fibers; C:F) were lower in the GA‐s muscle in females, and the C:F was lower in EDL when compared to males. Capillarity in other muscle groups did not differ by sex. Fiber areas generally did not differ by sex, and a smaller fiber area in females was only evident in EDL (Table 2). Females had a lower prevalence of type I (“red, slow oxidative”) and higher prevalence of type II (“white, fast glycolytic”) myofibers in SOL. No obvious differences were seen in other muscle groups.

**TABLE 2:** Muscle capillary and fiber type phenotypes in adult male and female mice

	Female	Male	*p* value
*C:F (#capillaries/#fibers)*			
Heart	*na*	*na*	*–*
GA‐d	1.62 ± 0.07	1.74 ± 0.05	0.35
GA‐s	1.13 ± 0.30	1.46 ± 0.05	<0.0001***
SOL	1.93 ± 0.07	2.05 ± 0.06	0.48
EDL	1.28 ± 0.06	1.46 ± 0.08	0.08
PLT	2.02 ± 0.09	1.99 ± 0.08	0.81
*Capillary Density (#/mm^2^)*			
Heart	2466 ± 46	2420 ± 70	0.98
GA‐d	732 ± 35	760 ± 23	0.49
GA‐s	389 ± 14	504 ± 10	<0.0001***
SOL	921 ± 26	928 ± 29	0.84
EDL	734 ± 32	742 ± 31	0.87
PLT	930 ± 31	915 ± 43	0.79
*Mean Fiber Area (μm^2^)*			
Heart	*na*	*na*	
GA‐d	2171 ± 79	2080 ± 57	0.35
GA‐s	2570 ± 68	2457 ± 45	0.17
SOL	2119 ± 63	2193 ± 85	0.48
EDL	1707 ± 46	2004 ± 99	0.008**
PLT	2191 ± 129	2229 ± 88	0.81
*Type 1 fibers (%)*			
Heart	*na*	*na*	
GA‐d	33.5 ± 1.0	33.1 ± 0.9	0.74
GA‐s	0.3 ± 0.1	0.2 ± 0.1	0.76
SOL	44.6 ± 1.5	50.6 ± 1.3	0.004**
EDL	16.1 ± 1.2	13.2 ± 0.9	0.06
PLT	41.8 ± 1.5	37.9 ± 1.8	0.11
*Type 2 fibers (%)*			
Heart	*na*	*na*	
GA‐d	66.7 ± 1.1	66.8 ± 0.8	0.90
GA‐s	99.7 ± 0.1	99.8 ± 0.1	0.72
SOL	55.6 ± 1.5	49.6 ± 1.2	0.004**
EDL	83.9 ± 1.2	86.8 ± 0.9	0.07
PLT	58.2 ± 1.5	62.1 ± 1.8	0.11

GA‐d, deep gastrocnemius; GA‐s, superficial gastrocnemius; SOL, soleus; EDL, extensor digitorum longus; PLT, plantaris; na, not applicable.

*n* = 14–18 (females) and *n* = 15–19 (males); sample sizes differed due to some samples not having adequate histology quality to enable scoring, or insufficient sample to conduct the analysis.

Values are means ± SEM.

** *p* < 0.01

*** *p* < 0.001.

#### 3.1.2 Gastrocnemius transcript patterns

**FIGURE 1:**
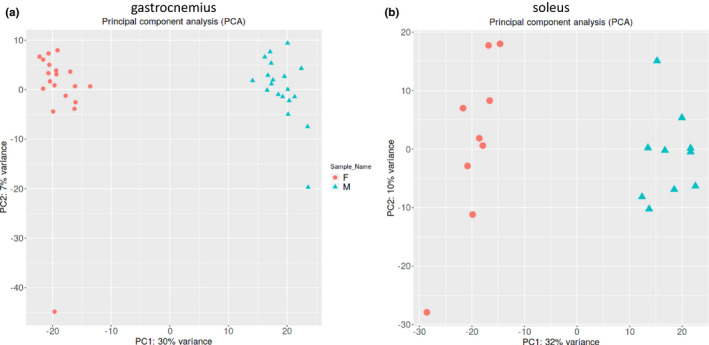
Principal components analysis (PCA) scores plot using all transcript data in female and male gastrocnemius (a) and soleus (b). Unbiased PCA multivariate statistical analysis evaluates variance in multiple dimensions for all transcripts, generating an individual score for each animal, represented by symbols (circles, females; triangles, males). These results show stark separation between males and females, indicating that the muscle transcriptome provides a distinct sex‐specific signature in both muscle groups

A large‐scale transcriptomics analysis was conducted in GA to compare differences between female and male mice in a mixed muscle context (*n* = 19/sex). The scores plot from an unbiased principal components analysis (PCA) multivariate statistical model using all transcripts reveals that variance in specific mRNA abundances can readily differentiate females vs. males (Figure 1). One female mouse was observed to differ from its counterparts (see symbol in lower left quadrant of the PCA scores plot). However, preliminary analysis of differentially‐expressed genes indicated that findings did not differ with or without inclusion of this mouse, and the female group as a whole were all well‐separated from male mice along the PC1 dimension even with this mouse included (Figure 1A). Thus, we included all mice in the final analyses presented.

There were 6362 transcripts expressed at significantly different levels comparing females and males (*p* < 0.05) (Supplemental Materials 1: https://data.nal.usda.gov/dataset/supplemental‐materials‐1‐adams‐et‐al‐skeletal‐muscle‐sexual‐dimorphism‐mice‐manuscript). A small subset of these transcripts are “sex‐specific” (Table 3). For instance, as expected the X‐chromosome‐linked *Xist* (inactive X specific transcripts) was absent in males, in contrast to females. Male‐specific transcripts included *Ddx3y* (DEAD box helicase 3, Y‐linked), *Eif2s3y* (eukaryotic translation initiation factor 2, subunit 3, structural gene Y‐linked), *Uty* (ubiquitously transcribed tetratricopeptide repeat containing, Y‐linked), and *Kdm5d* (lysine (K)‐specific demethylase 5D). Several genes were expressed at only trace amounts in one sex or another (defined here as ≤5% expression of the other sex) (Table 3). For instance, *Akr1cl* (aldo‐keto reductase family 1, member C‐like) expression was trace in males. Transcripts with only trace expression in females relative to males included: *DNAse1* (deoxyribonuclease I), *Slc30a2* (solute carrier family 30 (zinc transporter), member 2), *Sult1e1* (sulfotransferase family 1E, member 1), *Slc15a5* (solute carrier family 15, member 5), *Irx3os* (iroquois homeobox 3, opposite strand), *Gm29650* (unknown transcript), and *Themis3* (thymocyte selection associated family member 3).

**TABLE 3:** Sex‐specific* transcripts in gastrocnemius muscle of adult mice

Symbol	Gene name		Adjusted *p* value^b^
	FEMALE SPECIFIC*	Male, % of female^a^	
*Xist*	Inactive X‐specific transcripts	0%	**
*Akr1cl*	Aldo‐keto reductase family 1, member C‐like	5%	9.66E‐07
	MALE SPECIFIC*	Female, % of male^a^	
*Ddx3y*	DEAD box helicase 3, Y‐linked	0%	**
*Eif2s3y*	Eukaryotic translation initiation factor 2, subunit 3, structural gene Y‐linked	0%	**
*Uty*	Ubiquitously transcribed tetratricopeptide repeat containing, Y‐linked	0%	**
*Kdm5d*	Lysine (K)‐specific demethylase 5D	0%	**
*Themis3*	Thymocyte selection associated family member 3	2%	4.87E‐24
*Gm29650*	Unknown transcript	2%	2.29E‐27
*Irx3os*	Iroquois homeobox 3, opposite strand	2%	2.09E‐67
*Slc15a5*	Solute carrier family 15, member 5	2%	1.40E‐89
*Sult1e1*	Sulfotransferase family 1E, member 1	2%	5.52E‐23
*Slc30a2*	Solute carrier family 30 (zinc transporter), member 2	3%	1.48E‐164
*DNAse1*	Deoxyribonuclease I	4%	

^a^ Calculated as: % of female = 2^(log2 fold change)*100; % of male = 1/(2^(log2 fold change)*100), using females as comparator in iDEP.

^b^ False discovery rate‐adjusted p value from iDEP data output; Samples from *n* = 19/sex.

* “Specific” defined here as zero to ≤5% expression in one sex compared to the other.

** *p* values for binary comparisons with zero in one group are infinitely low.

Using the pathway analysis tools in iDEP, several patterns emerged for differentially‐expressed genes in GA. For GO Biological Processes and GO Cellular Component, pathways involving RNA management and protein translation appeared most predominant, with many transcripts lower in females compared to males (Table 4). Interestingly, transcripts associated with mitochondrial organization and structure were also reduced.

**TABLE 4:** GO Pathway analyses for sexually dimorphic transcripts in gastrocnemius muscle of adult mice

	Statistic	No. of genes	Adj. *p* value
*GO Biological Processes*			
mRNA processing	–5.9738	410	1.10E‐05
NcRNA metabolic process	–5.8177	443	1.20E‐05
RNA splicing	–5.4059	342	8.60E‐05
NcRNA processing	–5.164	339	0.00022
RNA splicing, via transesterification reactions	–4.6114	239	0.002
RNA splicing, via transesterification reactions with bulged adenosine as nucleophile	–4.6114	239	0.002
mRNA splicing, via spliceosome	–4.6114	239	0.002
Ribosome biogenesis	–4.575	274	0.002
Mitochondrion organization	–4.3379	438	0.0043
rRNA metabolic process	–3.9932	223	0.018
Proteasomal protein catabolic process	–3.9697	421	0.018
rRNA processing	–3.8898	194	0.025
Establishment of protein localization to organelle	–3.6677	344	0.048
*GO Molecular Function*			
Transmembrane signaling receptor activity	4.1155	407	0.016
GO Cellular Component			
Mitochondrial protein complex	–5.2368	247	9.60E‐05
Chromatin	–4.1676	500	0.0049
Spliceosomal complex	–4.087	181	0.006
Nuclear speck	–3.9497	362	0.0063
Organelle inner membrane	–3.6322	425	0.017
Inner mitochondrial membrane protein complex	–3.5979	113	0.017
Mitochondrial membrane part	–3.5206	212	0.017
Mitochondrial inner membrane	–3.4823	379	0.017
Nuclear chromosome part	–3.4756	478	0.017
Ribosome	–3.4028	221	0.022
Organellar ribosome	–3.3957	91	0.024
Mitochondrial ribosome	–3.3957	91	0.024
Catalytic step 2 spliceosome	–3.3556	82	0.026
Chromosomal region	–3.2403	242	0.027
Ubiquitin ligase complex	–3.2056	258	0.028
Golgi membrane	–3.1122	493	0.034
Nuclear chromatin	–3.0917	345	0.035
U2‐type spliceosomal complex	–3.0793	88	0.04
Endosomal part	–3.0756	344	0.035
Ribosomal subunit	–3.0172	186	0.04

Shown are pathways with FDR‐adjusted *p* < 0.05; transcript data from *n *= 19/sex; negative values indicate lower expression in females.

The Human Metabolome Database (HMDB) query returned no significantly altered pathways at adjusted *p* < 0.05. Nevertheless, transcripts associated with NADPH (HMDB00221), ammonia (HMDB00051) and phosphatidylethanolamine (PE) metabolism were returned as hits (*p* = 0.14; see Supplemental Materials 1: https://data.nal.usda.gov/dataset/supplemental‐materials‐1‐adams‐et‐al‐skeletal‐muscle‐sexual‐dimorphism‐mice‐manuscript). Heatmaps generated from the HMDB analysis highlighted sex differences in a variety of metabolic genes associated with these pathways. For instance, a survey of the NADPH metabolism heatmap (Supplemental Materials 1) revealed that *Hsd11b1* (hydroxysteroid 11‐beta dehydrogenase 1), *Cyp27a1* (cytochrome P450, family 27, subfamily a, polypeptide 1), and *H6pd* (hexose‐6‐phosphate dehydrogenase [glucose 1‐dehydrogenase]) had lower expression in females, by 45% (*p* = 5.83E‐60), 32% (*p* = 1.94E‐23), and 25% (*p* = 2.13E‐28), respectively. There were a variety of transcripts that were higher on average in female GA, but there at least two that stood out in magnitude: *Dhrs9* (dehydrogenase/reductase (SDR family) member 9) and *Duox1* (dual oxidase 1), which were ~3.3‐fold (*p* = 6.50E‐07) and ~1.8‐fold (*p* = 8.06E‐12) higher in females. The ammonia metabolism transcript heatmap (Supplemental Materials 1) highlighted several transcripts that were consistently higher across individual female mice compared to males: e.g., *Dctd* (dCMP deaminase; 38% increased, *p* = 0.022), *Maoa* and *Maob* (monoamine oxidase A and B; 20%, *p* = 1.7E‐09 and 38%, *p* = 6.23E‐19), *Padi2* (peptidyl arginine deiminase, type II; 56%, *p* = 3.62E‐28), *Ampd3* (adenosine monophosphate deaminase 3; 37%, *p* = 1.6E‐11), and *Cth* (cystathionase [cystathionine gamma‐lyase]; ~twofold, *p* = 4.22E‐18). For PE metabolism‐associated transcripts, (Supplemental Materials 1), an example of an altered transcript is *Pla2g5* (phospholipase A2, group V) with ~threefold higher expression in females compared to males. From the master transcript list in Supplemental Materials 1, family members *Pla2g4b* and *Pla2g4a* mRNAs were also significantly increased in females, by 23% (*p* = 0.004) and 21% (*p* = 4.6E‐06), respectively. In contrast, certain other PLA2‐encoding transcripts were significantly lower in female GA: e.g., *Pla2g6* (10% lower, *p* = 1.24E‐06), *Pla2g4e* (15% lower, *p* = 0.0003), *Pla2g7* (24% lower, *p* = 5.07E‐08).

To assess the rigor and reproducibility of the GA RNASeq findings, we compared our dataset to the only paper comparing male and female mixed‐muscle transcript profiles in multiple mice per group (Yang et al., 2006). In that publication, hamstring mRNA abundances were measured in >300 mice fed high fat (42% fat calories) using Affymetrix chip‐based technology, which reported on known (annotated) and non‐annotated transcripts (e.g., predicted genes, expressed sequence tags [ESTs]). With the technological advantage of whole transcriptome sequencing and the full murine genome annotation, the current experiment extends these prior results significantly. Through curation of annotated transcripts from the supplemental data in (Yang et al., 2006) that were statistically significant and differed by at least 5% between males and females, we successfully cross‐checked 1177 of the 6362 transcripts that were significantly different in the current experiment (representing 18.5% of the total) (Supplemental Materials 1: https://data.nal.usda.gov/dataset/supplemental‐materials‐1‐adams‐et‐al‐skeletal‐muscle‐sexual‐dimorphism‐mice‐manuscript). From this analysis, there was 95% concurrence of significantly different annotated transcripts between both studies, providing confidence that the transcripts identified herein are robust in terms of sexual dimorphism.

#### 3.1.3 Soleus transcript profiles

Soleus specimens were available from a subset of mice (*n* = 9 females, *n* = 10 males) and used to characterize transcriptomics patterns complementary to those identified in GA. The scores plot from an unbiased principal components analysis (PCA) multivariate statistical model using all transcripts reveals that variance in mRNA abundances can readily differentiate females vs. males (Figure 1B).

There were 4451 transcripts that were significantly different (adjusted *p* value <0.05) (see Supplemental Materials 1: https://data.nal.usda.gov/dataset/supplemental‐materials‐1‐adams‐et‐al‐skeletal‐muscle‐sexual‐dimorphism‐mice‐manuscript). A small subset of these transcripts is “sex‐specific” (Table 5) and essentially mimicked those found in GA (see Table 3). For instance, *Xist* was absent in males, as was *Tsix* (X (inactive)‐specific transcript, opposite strand). Male‐specific transcripts included *Ddx3y*, *Eif2s3y*, *Uty*, and *Kdm5d*. The *Akr1cl* transcript expression was trace (defined as <5% vs. the other sex) in males, consistent with GA results. Interestingly, several transcripts with only trace expression in females relative to males in the GA (see Table 3 and GA Results section) displayed a different pattern in SOL: e.g., *DNAse1* mRNA was similar between sexes, *Slc30a2m*, *Slc15a5*, and *Irx3os* in females were expressed at 26%, 16%, 50% of male levels, respectively, and *Sult1e1*, *Gm29650*, and *Themis3* were not detected in SOL for either sex.

**TABLE 5:** Sex‐specific* transcripts in soleus muscle of adult mice

Symbol	Gene name		Adjusted *p* value^b^
	FEMALE SPECIFIC*	Male, % of female^a^	
*Xist*	Inactive X‐specific transcripts	0%	**
*Tsix*	X (inactive)‐specific transcript, opposite strand	0%	**
*Akr1cl*	Aldo‐keto reductase family 1, member C‐like	0.5%	5.97E‐12
	MALE SPECIFIC*	Female, % of male^a^	
*Ddx3y*	DEAD box helicase 3, Y‐linked	0%	**
*Eif2s3y*	Eukaryotic translation initiation factor 2, subunit 3, structural gene Y‐linked	0%	**
*Uty*	Ubiquitously transcribed tetratricopeptide repeat containing, Y‐linked	0%	**
*Kdm5d*	Lysine (K)‐specific demethylase 5D	0%	**

^a^ Calculated as: % of female = 2^(log2 fold change)*100; % of male = 1/(2^(log2 fold change)*100), using females as comparator in iDEP.

^b^ False discovery rate‐adjusted p value from iDEP data output; Samples from *n* = 9 females and *n* = 10 males.

* “Specific” defined here as zero to ≤5% expression in one sex compared to the other.

** *p* values for binary comparisons with zero in one group are infinitely low.

Using the pathway analysis tools in iDEP, several patterns emerged for differentially‐expressed genes in SOL. For GO Biological Processes and GO Cellular Component, pathways involving RNA management and protein translation appeared most predominant, with many transcripts lower in females compared to males (Table 6). These trends are similar to those seen in GA (see above). The Human Metabolome Database (HMDB) query returned no hits and no significantly altered pathways at adjusted *p* < 0.05.

**TABLE 6:** GO Pathway analyses for sexually dimorphic transcripts in soleus muscle of adult mice (shown are pathways with FDR‐adjusted *p* < 0.05)

	Statistic	No. of genes	Adj. p value
*GO Biological Processes*			
mRNA processing	–5.6975	411	4.00E‐05
NcRNA metabolic process	–5.6044	437	4.00E‐05
RNA splicing	–5.1684	340	0.00028
NcRNA processing	–5.1202	334	0.00028
RNA splicing, via transesterification reactions	–4.5339	237	0.0029
RNA splicing, via transesterification reactions with bulged adenosine as nucleophile	–4.5339	237	0.0029
mRNA splicing, via spliceosome	–4.5339	237	0.0029
Ribosome biogenesis	–4.4658	271	0.0032
rRNA metabolic process	–4.1199	221	0.013
rRNA processing	–4.0174	192	0.01
*GO Molecular Function*			
Transmembrane signaling receptor activity	4.4982	457	0.003
Receptor regulator activity	4.2364	254	0.005
Receptor ligand activity	4.1539	231	0.005
G protein‐coupled receptor activity	3.7446	224	0.02
*GO Cellular Component*			
Nuclear speck	–4.318	363	0.0055
Spliceosomal complex	–4.1452	177	0.0073

Shown are pathways with FDR‐adjusted *p* < 0.05; transcript data from *n* = 9 females, *n* = 10 males; negative and positive values indicate lower and higher expression, respectively, in females.

#### 3.1.4 Muscle group specificity of sexual dimorphism in gene expression

**FIGURE 2:**
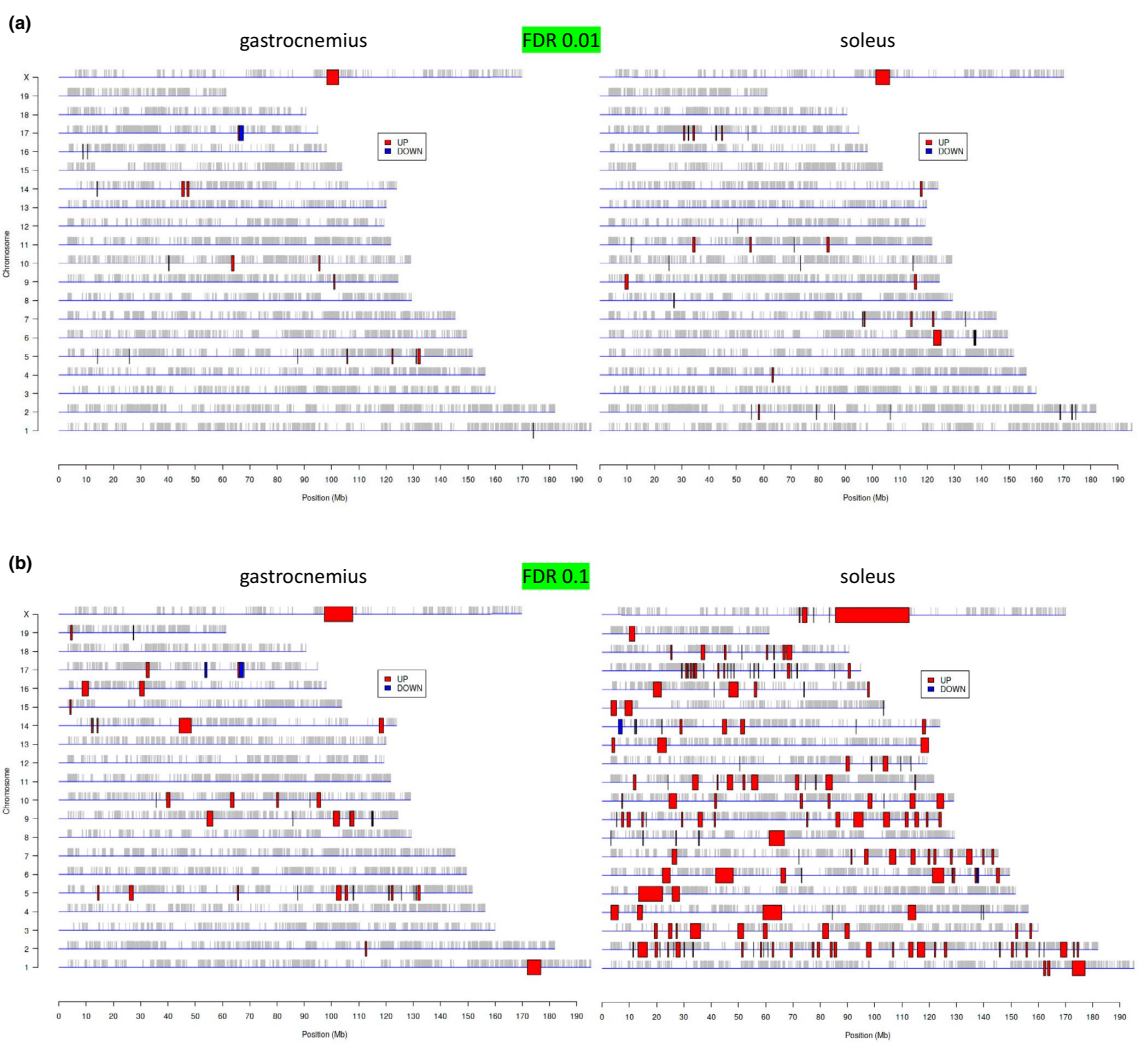
Mouse genome “hot spots” of differential gene expression when comparing skeletal muscle transcriptomes from adult male and female mice, in gastrocnemius (left panels) and soleus (right panels). (a) Differentially‐expressed genes at a stringent FDR *p* value cutoff of 0.01. Similar analyses were conducted with less stringent conditions at FDR *p* value 0.1 (b). Red and blue represent areas enriched with genes that displayed higher and lower expression, respectively, in female mice when compared to male mice. NOTE: the iDEP tool did not return data on the Y chromosome

**FIGURE 3:**
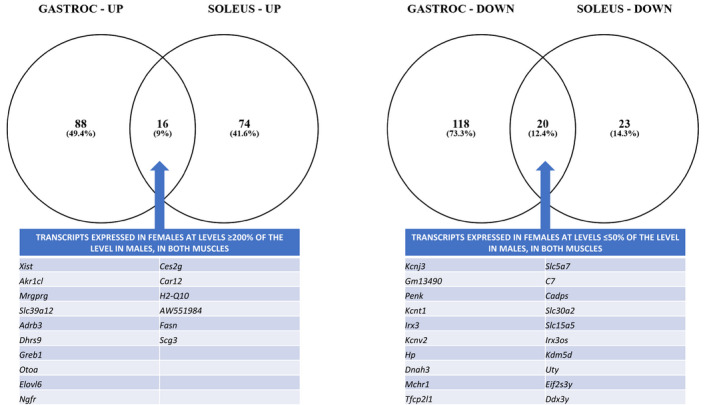
Venn diagrams depicting the number of muscle group‐specific and shared transcripts with expression levels in females that were (a) at least 200% of male levels or (b) were lower in females by at least 50%. Transcripts sharing patterns in both gastrocnemius and soleus muscles are provided in the embedded tables. The full list of transcripts with muscle‐specific or shared change patterns may be found in Supplemental Materials 1 (https://data.nal.usda.gov/dataset/supplemental‐materials‐1‐adams‐et‐al‐skeletal‐muscle‐sexual‐dimorphism‐mice‐manuscript). Diagrams and analyses were performed using Venny version 2.1 (https://bioinfogp.cnb.csic.es/tools/venny/)

Results in GA and SOL highlight significant sex effects on gene expression, with thousands of transcripts that differ significantly with very high to more subtle magnitude when comparing females and males. To evaluate which aspects are common versus specific to each muscle group, we made use of the PREDA (Position RElated Data Analysis) tool in iDEP. This method is based on a genome‐wide assessment of up‐ or downregulated gene regions, based on transcript information (Ferrari et al., 2011). This analysis revealed chromosomal “hot spots” of sexually dimorphic skeletal muscle gene expression in the mice. Figures 2A and B graphically display this phenomenon, with progressively less stringency in the genesets included in the analysis (FDR values of 0.01 and 0.1, respectively). At high statistical stringency (Figure 2A), only one region of the X chromosome circa 100 Mb displayed a consistent pattern of increased expression in females compared to males. This region encodes the *Xist* and *Tsix* transcripts and would thus be expected to be identified by the PREDA tool. With a lower FDR p value threshold of 0.1 (Figure 2B), the X chromosomal “hot spot” region seen in both GA and SOL broadened, and a few other shared regions became apparent: as an examples, at Chromosome 1 circa 174 Mb and at Chromosome 14 circa 118 Mb. Interestingly, substantial muscle group‐specific “hot spots” became more apparent: e.g., compare GA and SOL patterns on Chromosomes 3, 4, 6, 7, 11, 12, and 18 in which no specific regions of enhanced gene expression were observed in the former. A survey of Figure 3B makes it clear that even along the same chromosome the “hot spot” pattern can differ substantially between GA and SOL. These results are consistent with the concept that GA and SOL muscles share some factors that drive sexually dimorphic gene expression (especially on the X chromosome), but much of the regulation of this phenomenon is muscle‐specific.

Significantly different transcripts with expression patterns unique to muscle groups plus those that shared directional changes in both GA and SOL were considered using Venn analysis (see Supplemental Materials 1 for all transcripts included in the Venn diagrams: https://data.nal.usda.gov/dataset/supplemental‐materials‐1‐adams‐et‐al‐skeletal‐muscle‐sexual‐dimorphism‐mice‐manuscript). The most robust factors in terms of magnitude of difference between females and males is presented in Figure 3: these mRNAs were expressed in females at ≥200% (Figure 3A) or ≤50% (Figure 3B) that of males. Whether looking at the full list or this subset of transcripts, the results are consistent with the conclusions from the gene “hot spot” results above, in that they illustrate that sexually dimorphic gene expression is apparent in different muscles but that only a minority of transcript differences are shared between GA and SOL.

## 4 DISCUSSION

Many aspects of muscle function, development, and anatomy differ when comparing females and males but the relative contributions of genetics, environment, fitness, or other factors such as endocrine function remain to be fully clarified. Animal models enable control of diet, environment, and genetics in order to characterize sex differences in muscle, including studies across disparate muscle types. The latter has historically not gained much attention but is important considering the unique roles each muscle type plays in physical function and metabolism (e.g., the relative contributions of “oxidative” and “glycolytic” myofibers to metabolism). Herein, we present new data derived from mouse GA, SOL, EDL, and PLT that highlight muscle‐specific sexually dimorphic skeletal muscle anatomical phenotypes. Many sex‐associated differences were also seen in the muscle transcriptome, and with unique patterns that distinguished GA from SOL. Interestingly, when sex‐associated transcripts shared in both muscles were compared to published results from mice and humans, specific transcripts were identified that we propose represent canonical systems that mark or regulate innate sexual dimorphism in skeletal muscle.

While highly variable, the functional and anatomical skeletal muscle phenotypes clearly differ in females and males. In men, for instance, there are typically larger myofiber sizes/cross‐sectional areas (Bamman et al., 2003; Hoeg et al., 1985; Miller et al., 1993; Porter et al., 1985; Simoneau and Bouchard, 1989; Staron et al., 2000; Yasuda et al., 1985), and on average men display greater strength and speed (Bamman et al., 2003; Billaut and Bishop, 2012; Esbjornsson et al., 1993; Frontera et al., 1985; Miller et al., 1993; Sbriccoli et al., 2007; Wiecek et al., 2016; Yasuda et al., 1985). In females, there tends to be lower exercise‐associated muscle fatigue (Billaut and Bishop, 2012; Fulco et al., 1999; Hakkinen, 1994; Wiecek et al., 2016), a higher prevalence of “oxidative” fibers (e.g., type I, type IIa) and lower prevalence of “glycolytic” fibers (type IIx, type IIb) (Miller et al., 1993; Staron et al., 2000). Peak fat oxidation during graded aerobic exercise (expressed per fat‐free mass) is significantly higher in females when matched to males of similar body composition (see: (Chrzanowski‐Smith et al., 2021; Devries, 2016) and references therein). Nevertheless, sex differences in some muscle phenotypes appear to be context‐dependent. For instance, some have reported more muscle capillarity in women (Hoeg et al., 1985; Roepstorff et al., 2006) whereas others have not (Porter et al., 1985; Sjogaard, 1982). Furthermore, sex differences in fiber type prevalence are not always observed, and there is high interindividual variability (e.g., (Porter et al., 1985; Simoneau and Bouchard, 1989; Yasuda et al., 1985)). Finally, factors such as fitness, performance level, and age play a major role in muscle size, strength, metabolism, and fatigue. These most certainly lead to variability that make it more challenging to identify inherent sex‐specific differences in skeletal muscle. Our studies in male and female mice, tested in cohorts of littermates at the same age under a controlled housing and nutritional environment and in the absence of training, enabled a definitive assessment of innate sex‐associated differences in muscle size, myofiber characteristics, capillarity, and transcriptome across multiple muscle groups.

Muscle weight as measured by individual muscle weights or as summed weight was higher in males in the current study, a consistent finding in post‐weaned and adult mice (e.g., Denies et al., 2014; Fearing et al., 2016; Griffin and Goldspink, 1973; Melton et al., 2016; Rowe and Goldspink, 1969). The specific factors that drive this phenomenon remain to be established. In the seminal work of Goldspink and colleagues (Griffin and Goldspink, 1973; Rowe and Goldspink, 1969), it was found that total muscle weight is very tightly correlated with body weight across the lifespan in chow‐fed mice, even pre‐weaning when sex hormone influences are less in play. Herein, we opportunistically leveraged samples from past and ongoing studies utilizing a high‐fat feeding paradigm that induces diet‐induced obesity (DIO). In this context, we found that summed muscle masses were not correlated with terminal body weight in males, females or when sexes were combined (Supplemental Materials 1: https://data.nal.usda.gov/dataset/supplemental‐materials‐1‐adams‐et‐al‐skeletal‐muscle‐sexual‐dimorphism‐mice‐manuscript). However, this is most certainly due to the high body fat contribution to body weight, since there was a very strong correlation between summed muscle weights and DEXA‐measured lean body mass (*r* ^2^ = 0.67, *p* < 0.0001; Supplemental Materials 1). These results and those of Goldspink et al. in non‐obese mice (Griffin and Goldspink, 1973; Rowe and Goldspink, 1969) highlight that factors linked to body‐wide lean tissue growth drive muscle growth in a highly coordinated fashion regardless of sex; greater growth trajectories during development in males therefore largely explains their higher muscle mass.

As for fiber type distributions, only the SOL had a significant sex difference with a lower prevalence of type I fibers and higher relative amount of type II fibers in females. This contrasts somewhat with the conclusions of DeNies et al. who compared muscle fiber type in SOL and PLT of male and female mice fed a control diet or an extreme high‐fat diet (HFD, 60% of energy) for 1 year (Denies et al., 2014). In the latter study, no significant sex or sex × diet effects were reported for PLT muscle, and no sex differences in SOL myofiber percentage were observed in mice fed the control diet. Opposite to our findings, they reported a greater prevalence of type I fibers in HFD female SOL compared to HFD males. This was due to a drop in type I fibers following in HFD males only. The reasons for differences across the studies is not clear but might be due to the disparate diet paradigms (e.g., our mice were fed a 45% fat diet for ~13 week), which would have led to large differences in adiposity or and/or insulin resistance when comparing studies. In males, type I fiber prevalence was inversely correlated with a measure of adiposity (Denies et al., 2014). In humans, adiposity and/or related sequelae such as insulin resistance inversely correlate with prevalence of type I fibers (e.g., (Hickey et al., 1995; Kriketos et al., 1996; Marin et al., 1994)), but whether or not this differentially impacts males and females or applies in a mouse model remains to be established. A caveat to interpreting myofiber type based on the classic histology methods used herein is that sex differences in distinct fiber sub‐populations or their functional components cannot be fully discerned (Schiaffino, 2018). Looking at myosin heavy chain (Myh) component mRNA patterns, expression of the fast‐twitch glycolytic type IIx marker *Myh1* was significantly lower in female SOL by 50%, but this mRNA was unchanged in GA. The oxidative slow‐twitch type I mRNA *Myh7* was significantly higher by 34–75% in both SOL and GA. In SOL and GA, mRNA markers typically associated with muscle regeneration were significantly higher in females (e.g., *Myh3* [by 50–60%] and *Myh8* [37% to 2‐fold]). Other myofiber subtype markers in SOL (e.g., *Myh2* [fast‐twitch glycolytic type IIa], *Myh4* [fast‐twitch type IIb]) were not significantly altered by sex, in contrast to GA where these transcripts were increased and decreased, respectively. Taken together, these observations and those in the literature strongly support the principles that: (a) in mice and humans, females and males display innate differences in skeletal muscle fiber sub‐types and myosin biology with regulation that is context‐dependent, and (b) sexual dimorphism patterns for myofiber type marker *Myh* transcripts are muscle group‐specific and do not always track histological findings.

In addition to fiber type we measured fiber area across multiple muscles, and based on the literature we expected to detect larger fibers in male mice (Fearing et al., 2016; McHale et al., 2012; Melton et al., 2016; Rowe and Goldspink, 1969). However, only the EDL myofiber area was larger in males compared to females, with no differences in GA‐d, GA‐s, SOL, and PLT. At this time, there is no clear explanation for this unanticipated finding except to note that prior studies examining cross‐sectional areas were conducted in non‐obese mice fed standard diets. A plausible hypothesis is that HFD, obesity, or sub‐optimal metabolic health modify muscle phenotypes in a way that diminishes sex differences (e.g., through lowering fiber area in males). This idea is speculative but may be tested in future studies that characterize myofibers in obese and non‐obese conditions, with and without metabolic dysfunction. As with myofiber area, there were few gross differences in capillarity with the exception of significantly higher (30%) capillary indices (C:F ratio and capillary density [number/mm^2^]) in type II fiber‐enriched superficial gastrocnemius (GA‐s) in males. Due to larger muscle mass in males it follows that absolute total skeletal muscle capillary volume in the body would have been larger when compared to females, even in the absence of a difference in capillary density. Consistent with the generally similar capillary indices in most muscle groups, transcriptomics pathway analyses (discussed in detail below) did not reveal any obvious sex differences in expression patterns related to neovascularization or capillarity pathways. Overall, the findings suggest that: (a) per unit of tissue volume the muscle capillary blood flow capacity in DIO mice is not innately different between sexes in most muscle groups, and that (b) there are muscle group‐specific regulators that can modify sexual dimorphism in capillarity. Considering the sexual dimorphism of capillary indices in GA‐s, future studies could focus on within‐GA differences in gene or protein expression to identify potential molecular regulators in the GA‐s.

Sexual dimorphism in muscle phenotype was also determined at the level of the transcriptome in whole GA and SOL. Relatively few studies have addressed this question in mice (Yang et al., 2006; Yoshioka et al., 2007) or humans (Liu et al., 2010; Maher et al., 2009; Roth et al., 2002; Welle et al., 2008). To our knowledge, sex‐associated differences have not been evaluated using RNASeq technology, or across disparate muscle groups. The first take away message is that sexual dimorphism in gene expression is profound: there were >6000 and >4000 significantly different transcripts in GA and SOL, representing 40% and 25% of all detected mRNAs, respectively. In the seminal 2006 study of Yang et al. (Yang et al., 2006) who studied mixed‐muscle type hindlimb transcripts by microarray in >300 mice fed a 42% fat diet 16 week, ~66% of detected transcripts were significantly differentially‐expressed, most with modest differences that did not achieve the oft‐used twofold difference cutoff (similar to our findings). In terms of significantly different transcripts that are “female biased” versus “male biased,” there appears to be a relatively even split in mouse muscle: e.g., 2672/2181 (female/male) in hindlimb (Yang et al., 2006) and 3181/3181 and 2173/2278 in GA and SOL, respectively herein. When we compared differentially‐expressed annotated (“known identity”) transcripts from the previous hindlimb microarray data (Yang et al., 2006) with our more complete GA mRNASeq results herein, there was 95% concurrence. This adds confidence to our interpretations and supports the concept that significant sex‐associated gene expression differences are inherent to skeletal muscle.

Pathway analyses can help identify systems that are potentially more engaged in one sex vs. the other. These interpretations must be tempered by the fact that mRNA patterns do not fully reflect protein levels, post‐translational modifications or signaling events that drive function. A common theme from GO pathway analysis of the current datasets is that transcripts involved with ribosomes, RNA processing, splicing and protein translation were generally reduced in female skeletal muscle (GA and SOL). Interestingly, ribosome biosynthesis/assembly and translation pathways were also identified in comparing female and male hindlimb in HFD mice (Yang et al., 2006). A 2007 study that employed serial analysis of gene expression (SAGE) in GA samples (pooled samples, *n* = 1/sex) from chow‐fed mice also noted differential expression of genes related to transcription/translation/ribosomal protein processes (Yoshioka et al., 2007). However, in both studies female expression of the pathways was higher than males, which contrasts to our findings. In an experiment comparing vastus lateralis of 15 men and 15 women using gene chip technology (Welle et al., 2008), men showed pathway enrichment for transcripts related to protein translation and initiation and ribosomal proteins (“RNA binding”), similar to patterns in our study. We noted that the DEAD‐box helicase 3, Y‐linked (*Ddx3y*) mRNA was male‐specific in both GA and SOL, and interestingly the X chromosome homolog *Ddx3x* was significantly higher by ~42–48% in female muscle. The DDX3 family of proteins take part in “RNA unwinding” and several other RNA‐related activities such as nuclear export and initiation of translation (Kotov et al., 2017; Mo et al., 2021). The transcription initiating factor *Eif2s3y* mRNA was also a “male‐specific” transcript in both muscles. Thus, a common theme emerges from our data and the extant literature that there is sexual dimorphism in muscle ribosome biology, RNA processing transcription, and translation, but directionality (male versus female) of specific aspects are context‐ and sometimes muscle group‐specific.

By comparing sexually dimorphic gene expression patterns in GA and SOL by Venn analysis and chromosome “hot spot” evaluations, it is clear that some regulation was shared but that most differentially‐expressed transcripts were muscle group‐specific. That sexual dimorphism of gene expression is tissue‐specific is well‐established (e.g., see comparisons of brain, perigonadal adipose, liver, and hindlimb muscle (Yang et al., 2006)), but to our knowledge has not been evaluated previously across different muscle beds. Our results, indicate that there are modifiers of sex effects that are muscle group‐dependent, but their characterization awaits future studies focused on multi‐muscle comparisons of gene and transcriptional regulators: e.g., differential epigenetic patterns, sex hormone signaling, and gene regulatory elements. One anticipated finding was that in both GA and SOL, there was sex‐specific engagement of the X chromosome inactivation (XCI) machinery. This system involves female‐specific expression of the long, non‐coding regulatory RNA *Xist* that serves to strongly attenuate gene expression from the largely inactivated X chromosome (Xi) in comparison to the activated X chromosome (Xa), thereby dampening the X chromosome gene expression dosage that would otherwise greatly exceed that of males who carry just one X copy (Lucchesi, 2018; Pontier and Gribnau, 2011). The chromosome “hot spot” analysis of sexually dimorphic gene expression identified the X chromosome region containing Xist as a shared component in GA and SOL. While *Xist* is transcribed from the Xi, the *Tsix* transcript is expressed from the Xa and counters the activity and expression of Xist. Interestingly, *Tsix* expression was detected in SOL but not GA. It is interesting to speculate that a differential balance of *Xist* and *Tsix* expressions underlies some aspects of muscle group‐specific sexual dimorphism.

The comprehensive GA and SOL transcript data provide a rich source of information to consider groups of genes that are regulated in a sex‐ and muscle‐specific manner in mice, and as previously noted these results showed excellent alignment with a previous microarray study in DIO mice (Yang et al., 2006). By testing across four tissues, Yang et al. identified 27 transcripts that were sexually dimorphic and shared directionality in all of the tissues (see Table S7 in the supplemental materials in (Yang et al., 2006)), suggestive of genes that are central to driving sex‐associated differences across disparate tissue types. We cross‐checked that list with our data for GA and SOL, reasoning that transcripts with consistent patterns across both muscle groups and the tissues reported previously (Yang et al., 2006) will be some of the most robust in terms of involvement with multi‐tissue sexual dimorphic regulation in mice. This revealed five transcripts highest in males (*Ddx3y, Eif2s3y, Kdm5d* [aka *Jarid1d*]*, Lrg1* [leucine rich alpha‐2‐glycoprotein 1]*, Asb5* [ankyrin repeat and SOCs box‐containing 5]) and six transcripts highest in females (*Polg* [polymerase (DNA directed), gamma]*, Hacd1* [3‐hydroxyacyl‐CoA dehydratase 1; aka *Ptpla], Cnpy2* [canopy FGF signaling regulator 2; aka *Tmem4*]*, Oas2* (2'‐5'‐oligoadenylate synthetase 2)*, Kdm6a* [aka *Utx*]*, Gbp3* [guanylate binding protein 3]). One can also consider if there are transcripts reflecting cross‐species canonical events related to sexual dimorphism in skeletal muscle. Using the transcripts above, plus our list of sexually dimorphic transcripts (“sex‐specific” genes from Tables 3 and 5 that were shared in GA and SOL), and our lists of the most robustly different transcripts (Figure 3) as a foundation, we compared against transcripts reported as different in men and women from comprehensive skeletal muscle transcriptomics experiments: e.g., (Welle et al., 2008) (using their Table 2 that included transcripts with ≥twofold change, *p* < 0.0001 that excluded X‐ and Y‐associated mRNAs, and the entire transcript list in their supplemental data) and (Maher et al., 2009) (using their Supplementary Table 1 containing differentially‐expressed genes they defined as fold change at least 1.2). Using this strategy, we identified four genes consistently expressed more abundantly in female skeletal muscle: *Xist*, *Kdm6a* (aka *Utx*), *Grb10* (growth factor receptor bound protein 10), and *Oas2*. At least six transcripts were expressed more abundantly in male skeletal muscle: *Ddx3y*, *Kdm5d*, *Irx3* (iroquois homeobox 3), *Wwp1* (WW domain containing E3 ubiquitin protein ligase 1), *Aldh1a1* (aldehyde dehydrogenase 1 family member A1), and *Cd24a* (CD24a antigen). Using a list of differentially‐expressed genes in human‐derived female and male myotubes (Davegardh et al., 2019), in addition to *Xist* the mRNA for *Rps4x* (ribosomal protein S4 X‐linked) was higher in females and this pattern shared in the murine datasets and the human results of Welle et al. (2008) (but not reported by Maher et al. (2009) as a transcript differentially‐expressed by at least 1.2‐fold). We propose that this group of genes represent a subset of canonical markers of innate sexual dimorphism in mammalian skeletal muscle (Table 7). A survey of Table 7 transcripts highlights that the majority‐‐if not all‐‐involve gene regulation, ribosomal biology, cell growth/differentiation/proliferation pathways. Since comparisons across studies are limited by differences in technologies, gene coverage, numbers of known versus non‐annotated transcripts, definitions of significance, experimental conditions, etc., the current list of proposed “canonical” genes is most certainly only a subset of a larger group that will be discovered to be involved with inherent sexual dimorphism in skeletal muscle.

**TABLE 7:** A subset of potential canonical transcripts marking and/or regulating sexual dimorphism of skeletal muscle in mice and humans*

Symbol (mouse chromosome; human chromosome)	Gene name	Function and Comments
*Higher Expression In Females*		
*Xist* (X, 46.15 cM; Xq13.2)	Inactive X‐specific transcripts	Non‐coding RNA that interacts in cis with the Xi to attenuate expression of most Xi genes; key to XCI
*Kdm6a* (X, 13.45 cM; Xp11.3)	Lysine (K)‐specific demethylase 6A (aka *Utx*)	Typically escapes XCI; alters histone methylation status (demethylation of H3K27me3, H3K4me3); regulator of developmental genes; activated by α‐KG, inhibited by fumarate, succinate, 2‐HG, hypoxia; role in gene expression regulation (activation?); checkpoint mediator/tumor suppressor; cell growth and proliferation
*Grb10* (11, 7.15 cM; 7p12.1)	Growth factor receptor bound protein 10	Phosphorylation target of mTORC1, regulator of receptor tyrosine kinases: inhibits insulin signaling by blocking IRS/phosphor‐IR interaction; maternally‐imprinted gene in embryo/fetus as governor to growth; regulated by differentially‐methylated region in promotor; governor on muscle fiber numbers
*Oas2* (5, 60.64 cM; 12q24.13)	2′‐5′ oligoadenylate synthetase 2	Interferon‐responsive “antiviral” gene; increased muscle expression in auto‐immune Juvenile Dermatomyositis that affects muscle
*Rps4x* (X, 45.20 cM; Xq13.1)	Ribosomal protein S4 X‐linked	40S and 18S ribosome protein, important to ribosome packaging and structure; in *Xist* genomic region; escapes XCI in humans but not mice and so primarily expressed by Xa in female mice and both Xa and Xi in humans
*Higher Expression In Males*		
*Ddx3y* (Ypter; Yq11.221)	DEAD box helicase 3, Y‐linked	RNA helicase; role in cell growth, stem cell‐derived cardiogenesis; testis spermatogenesis;
*Kdm5d* (Ypter; Yq11.223)	Lysine (K)‐specific demethylase 5D (aka *Jarid1d*)	Alters histone methylation status (demethylation of H3K9me3, H3K4me[1,2,3]); activated by α‐KG, inhibited by fumarate, succinate, 2‐HG, hypoxia; role in gene expression regulation; checkpoint mediator/tumor suppressor; cell growth and proliferation
*Irx3* (8, 44.55 cM; 16q12.2)	Iroquois homeobox 3	Vertebrate embryogenesis; promoter binds SREBF2 and PPARG in adipose; expression regulated by *FTO*, implicated in metabolic homeostasis
*Wwp1* (4, 7.56 cM; 8q21.3)	WW domain containing E3 ubiquitin protein ligase 1	Cell proliferation, K63 ubiquitylation of p53 promotes p53 stability‐movement to cytosol/limits p53‐induced gene expression; apoptosis stimulation; tumorigenesis promoter
*Aldh1a1* (19, 13.91 cM; 9q21.13)	Aldehyde dehydrogenase 1 family member A1	Conversion of aldehydes to carboxylic acids derivatives, NADP‐dependent; retinaldehyde to retinoic acid conversion, regulating RARs/RXRs; tumorigenesis promoter
*Cd24a* (10, 23.01 cM; 6q21)	CD24 antigen	JAK, PI3K‐Akt, Ras‐MAPK actions; metastasis and tumorigenesis promoter; androgen‐stimulated expression

* See Discussion; transcripts are those significantly different in female versus male mouse gastrocnemius (GA) and soleus (SOL) in the current study, and with consistent patterns when cross‐curating against available skeletal muscle datasets from humans (Maher et al., 2009; Welle et al., 2008). XCI, X chromosome inactivation; Xa, active X chromosome; Xi, inactivated X chromosome; α‐KG, alpha‐ketoglutarate; 2‐HG, 2‐hydroxyglutarate; JAK, Janus family of protein kinases; MAPK, mitogen‐activated protein kinase; PI3K‐Akt, Phosphatidylinositol‐3‐Kinase and Protein Kinase B; PPARG, peroxisome proliferator‐activated receptor ϒ; RAR, retinoic acid receptor; RXR, retinoid X receptor; Ras, guanine nucleotide binding protein; SREBF2, sterol regulatory element‐binding transcription factor 2.

Our experiments were not designed to directly test the relative contribution of hormone regulation to female versus male muscle phenotypes. For instance, we report on one timepoint, and so any estrogen or androgen roles during development stages leading up to adult phenotype could not be evaluated. Nevertheless, it is useful to survey our proposed canonical genes (Table 7), “sex‐specific” transcripts (Tables 3 and 5), and the most robust differentially‐expressed transcripts (Figure 3), with respect to the potential for estrogen or androgen regulation. With the exception of *Greb1*, no genes from this survey are included in the comprehensive list of genes containing estrogen receptor binding sites in humans and mice (Bourdeau et al., 2004; Lin et al., 2007). These findings are consistent with the hypothesis that the majority of sexual dimorphism in muscle gene expression “at maintenance” during adulthood comes about through estrogen‐independent mechanisms. Supporting this postulate further are data from a fish model looking at sex‐dependent transcript differences in tissue including muscle: there were 480+ transcripts higher in females compared to males, but only 19 of these were estrogen‐induced in hormone‐treated males (Anderson et al., 2020). In contrast, comparing our gene subset against comprehensive lists of transcripts modified in androgen receptor knockout mouse gastrocnemius (MacLean et al., 2008) or genomic regions with one or more Chip‐positive androgen response element sites in human myoblasts treated with 5α‐dihydrotestosterone (Wyce et al., 2010), we identified 12 genes that are *potentially* androgen‐regulated (*Uty*, *Slc30a2*, *Grb10*, *Rps4x*, *Irx3*, *Aldh1a1*, *Cd24a*, *Dhrs9*, *Elovl6*, *Dnah3*, *Mchr1*, and *Cadps*). Altogether, our analysis points to a plausible model to explain patterns of sex‐associated transcriptomics differences in mice and humans, which involves an initial trigger by X inactivation in females (*Xist*) and Y activation in males (*Ddx3y*) and coincident engagement of the demethylase epigenetic regulators *Kdm6a* (in females) and *Kdm5d* (in males). This, in turn, leads to a cascade of events that are complemented by modulation of select androgen‐response regions of the genome.

In conclusion, comprehensive phenotyping of skeletal muscle in the controlled environment of a DIO mouse study revealed distinct, sexually dimorphic aspects reflected in muscle weights (highest in males), capillarity (higher density in male GA‐s only), myofiber size (highest in male EDL only), and fiber type (higher and lower prevalence of type I and type II, respectively, in female SOL). Most striking were differences in transcript patterns in females and males, with thousands of mRNAs differentially‐expressed and only a minority shared across GA and SOL. The latter reflects muscle type‐specific regulation of gene expression. Transcriptomics revealed a subset of shared pathways that differed between males and females, most notably sex‐dependent differences in expression of genes involved with ribosome biology and transcription and processing of RNA. In addition, a comparison of our transcriptomics results to the available literature comparing male and female muscle in mice and humans identified 11 mRNAs that consistently display sex‐dependent expression differences, suggesting roles for these genes in marking, triggering, or regulating sexual dimorphism. We conclude that innate differences in adult male and female skeletal muscle are significant and manifest in muscle group‐specific ways, including differences in large numbers of expressed genes.

## CONFLICT OF INTEREST

S.H. Adams is founder and principal of XenoMed, LLC, which is focused on research and discovery unrelated to the studies herein.

## AUTHOR CONTRIBUTIONS

Study design (JOR, KOM, IMO, SHA); Conducted research (JOR, KOM, JMR, TT); Analyzed data (JOR, KOM, SVC, IMO, SHA); Provided key experimental infrastructure and assets (KCKL, IMO, SHA); Wrote manuscript (SHA, with key input from JOR, KOM, IMO); all authors helped interpret results, edited the manuscript drafts, and approved the final paper.

## Supporting information



Supplementary Material
